# Data-driven de novo design: revolutionizing super-adhesive hydrogels for underwater applications

**DOI:** 10.7150/ijbs.128485

**Published:** 2026-01-27

**Authors:** Ying Cen, Xue-miao Liu, Zhi-Qiang Song, Hong-Juan Fang, Xing Wang

**Affiliations:** 1 Aviation General Hospital, Beijing 100012, China.; 2 Beijing National Laboratory for Molecular Sciences, Institute of Chemistry, Chinese Academy of Sciences, Beijing 100190, China.; 3 University of Chinese Academy of Sciences, Beijing 100049, China.

Hydrogel adhesives possess characteristics such as flexibility and biocompatibility, which make them highly promising in fields like biomedical engineering and flexible electronics. However, their inferior underwater wet adhesion and limited stability severely restrict their practical development 1, 2. Traditional hydrogel design relies on trial-and-error methods. Due to the variety and quantity selection of building units (such as monomers) and their arrangement combinations, a vast design space is formed. Moreover, the complex multi-scale structure-performance relationships of soft materials (interwoven molecular weak interactions and thermal fluctuations) hinder precise theoretical prediction 3, 4. While natural adhesion proteins (biological soft tissues) exhibit exceptional wet adhesion, mining and synthesizing their sequence patterns remains a core challenge. In recent years, AI has transformed the material design paradigm, and particularly, data mining and machine learning-driven biomimetic hydrogel design have shown significant potential 5. The Gong team 6 proposed an innovative interdisciplinary framework bridging biological sequence mining (biology), functional monomer design (chemistry), and predictive modeling (computer science). This strategy achieved precise design of super-adhesive hydrogels: initial synthesis yielded up to 147 kPa underwater adhesion strength, and optimization pushed it over 1 MPa—filling the industrial underwater adhesion gap (Figure 1a).

This method starts with natural adhesion protein data mining: 24,707 sequences were collected from NCBI (keyword: "adhesion protein"), covering 3,822 species (average length: 300-500 amino acids; Figure 1b). After screening 200 species with the most adhesion proteins, the conserved sequences of each species were obtained through Clustal Omega multiple sequence alignment 7. 20 amino acids were classified into 6 physicochemical categories (hydrophobic, nucleophilic, acidic, cationic, amide, aromatic), and adjacent functional groups showed specific pairing preferences (Figure 1c). Based on this, 6 functional monomers (BA, HEA, CBEA, ATAC, AAm, PEA) were selected to mimic corresponding amino acid functions. For example, HEA for nucleophilic amino acids 8, CBEA for acidic amino acids 9, and ATAC for cationic amino acids 10 (Figure 1d). A one-pot free radical copolymerization reaction using Irgacure 2959 as the photoinitiator was carried out: The precursor solution was transferred to a glove box to remove oxygen, then poured into a reaction vessel consisting of a pair of glass plates, and exposed to ultraviolet light at room temperature for 8 hours to form an organic gel, then the 180 data-driven hydrogels were synthesized (Figure 1e). The test results showed that the adhesion strength of 16 gels exceeded 100 kPa (with G-042 reaching 147 kPa). Finally, based on 180 hydrogel data, after comparing 9 machine learning models, the Gaussian process and random forest regression were selected to optimize the formula, and after 3 iterations, three gels with R1-max, R2-max, and R3-max were obtained. The underwater adhesion strength of all three gels exceeded 1 MPa (Figure 1f), which is an order of magnitude higher than the initial gel and significantly superior to existing underwater adhesives.

The environmental dependency test indicates that the adhesion strength (*F*_a_) increases with the increase of loading force and contact time until it reaches a plateau (the enhancement of interface contact and the drainage effect jointly contribute to this). The *F*_a_ of R1-max in physiological saline (glass substrate) exceeds 1 MPa and maintains stable performance after 200 cycles of adhesion; the adhesion strengths of the three gels are similar in artificial seawater; R2-max performs the best in deionized water and exhibits cavitation when detaching (Figure 1g). In practical applications, R1-max can adhere the rubber duck to the seaside rocks and withstand tidal impacts (Figure 1h), and R2-max can instantly seal the high-pressure leakage of a 3-meter-high water pipe with a 20 mm diameter hole**—**demonstrating its utility in emergency infrastructure repair. Additionally, the subcutaneous implantation experiment in mice showed no obvious inflammatory response or fibrosis after 2 weeks, confirming its suitability for biomedical applications like surgical wound closure (Figure 1i).

The Gong team, through the mining of natural protein databases and the optimization of AI models, proposed an integrated strategy of data mining, biomimetic design, and machine learning, enabling the hydrogel to reproduce the sequence heterogeneity and functional distribution of natural adhesion proteins, effectively eliminating the interference of interface water molecules, and achieving an underwater adhesion strength of over 1 MPa. This method breaks through the limitations of traditional trial-and-error and provides a revolutionary tool for the rational design of biomimetic hydrogels. In the future, challenges such as the diversity of functional monomers (e.g., monomers mimicking sulfur-containing amino acids like cysteine to introduce disulfide bond cross-linking) and precise regulation of polymer sequences need to be addressed to promote its practical applications in deep-sea exploration and biomedicine.

## Figures and Tables

**Figure 1:**
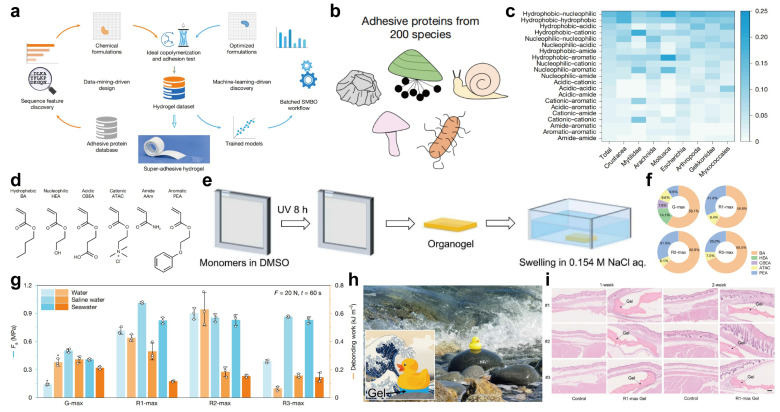
Scheme of Data-driven de novo design of super-adhesive hydrogels. (a) Data-driven de novo design of underwater adhesive hydrogels. (b) The amino acids source of synthetic biomimetic formulas. (c) Pairwise frequency distribution of the 21 functional class pair types along encoded sequences, shown for the entire dataset and for eight representative species, shown along the horizontal axis, categorized by their biological classifications in the database. (d) Chemical structures of six functional monomers, each representing one of the six functional classes of amino acids. (e) Schematic diagram illustrating the hydrogel fabrication procedure via free radical copolymerization. (f) Formulations of the three optimized gels (R1-max, R2-max, R3-max). (g) *F*_a_ on glass substrate in deionized water, normal saline and artificial seawater (0.7 M NaCl) for hydrogels equilibrated in the corresponding solutions. The asterisk on G-max indicates cohesive failure during testing. Error bars represent the standard deviation of N = 3 measurements. (h) Photographic images of R1-max adhering a rubber duck to a seaside rock, withstanding ocean tides. (i) Histopathologic profiles of tissues in contact with the R1-max hydrogel over time.

## Abbreviations

NCBI: National Center for Biotechnology Information
BA: Benzyl Acrylate
HEA: Hydroxyethyl Acrylate
CBEA: Carboxybenzyl Ethyl Acrylate
ATAC: Acryloyloxyethyl Trimethyl Ammonium Chloride
AAm: Acrylamide
PEA: Phenethyl Acrylate
AI: Artificial Intelligence
